# Science of recovery in schizophrenia research: brain and psychological substrates of personalized value

**DOI:** 10.1038/s41537-017-0016-6

**Published:** 2017-03-29

**Authors:** Kiyoto Kasai, Masato Fukuda

**Affiliations:** 1 Department of Neuropsychiatry, Graduate School of Medicine, The University of Tokyo, 7-3-1 Hongo, Bunkyo-ku, Tokyo 113-8655 Japan; 2 Department of Psychiatry and Neuroscience, Gunma University Graduate School of Medicine, 3-39-22 Showa, Maebashi, Gunma 371-8511 Japan

Although schizophrenia was first described as dementia praecox by E. Kraepelin more than a hundred years ago, assuming a neurodegenerative origin, neurodevelopmental hypothesis of schizophrenia ^1^ has been established by the late 20th century. In the last decade, clinical staging model of schizophrenia ^2^ has been increasingly recognized and early intervention is regarded as a promising strategy. The fact that early intervention for psychosis involves providing treatment in the critical period, i.e, at around the onset of the condition, has reminded us of how little we know about the developmental neuroscience of adolescence and how important it is to incorporate knowledge of adolescent brain development into schizophrenia research.^3^ Human adolescence is much longer than that in non-human primates, and is the stage of life in which the cerebral neocortex matures, thereby promoting human-specific self-regulation.^3^ Schizophrenia might be reconsidered as a developmental disorder of self-regulation in adolescence.

Major outcomes indicating clinical remission or recovery in schizophrenia have been symptomatology, neurocognition, and objective social functioning. However, the concept of “personal” recovery,^4^ tightly coupled with subjective well-being, has recently been emerging from the user’s point of view for providing a life worth living (without necessarily having a clinical recovery), which is about building a life that is satisfying, fulfilling, and enjoyable. Could personal recovery, a subjective (first-person) concept, be solely pursued through spiritual, ethical, and human rights/social capital approaches? Would the neuroscientific understanding of personal recovery be impossible? We believe it is possible. We should make efforts to scientifically characterize the conceptual framework of personal recovery, so that users, family members, caregivers, and professionals can understand and contribute to the users’ personal recovery and subjective well-being.

How do we achieve this? First, we should scientifically redefine a person’s “value.” Here, “value” refers to individuals’ way of facing the world, which both consciously and unconsciously drives their long-term actions throughout the course of their lives. While childhood is associated with trans-generational, passive incorporation of parental values, adolescence is characterized by social interactions with peers. Through such influences, a person’s value is internalized and personalized to become “personalized value,” on which the person can rely to actively make decisions on his/her own life in the long term.

To understand the neural basis of “personalized value,” its development can be modeled as the psychological process in which adolescents acquire the ability to control the conflict between learned value and actual behavior by using self-regulation, including meta-cognition and language (inner-speech). Here, we should consider the “real-world” as the field where an individual develops a personalized value to live his/her own life. We may hypothesize a spiral model where active interactions with the real-world influence value development, which then shapes patterns of action in life, in turn inducing plasticity in the brain circuit. Furthermore, use of a life-course epidemiologic approach is important to understand how personalized value is developed in adolescence and how it influences later life. By integrating the brain, real-world, and life-course approaches, we will be able to deepen the conceptual framework of personalized value and then develop psychosocial intervention strategies to cultivate it.

Schizophrenia is a disorder with adolescent onset for which functioning in the real world may have a long-term impairment. We can incorporate the concept of “personalized value” into schizophrenia research by combining the areas of developmental psychology and neuroscience in adolescence with developmental psychopathology in schizophrenia. This approach will be important to scientifically conceptualize and realize personal recovery, which may also be important to understand the concepts of “person-centered” approach and “democratizing” clinical research.^5^ Thus, schizophrenia research is in the frontline of the value-based approach,^6^ which has been recently proposed in the “beyond evidence-based medicine” approach.
